# Supplementary material for: Two New Phenolic Glycosides with Lactone Structural Units from Leaves of Ardisia crenata Sims with Antibacterial and Anti-Inflammatory Activities
Source: Molecules. 2022 Jul 31;27(15):4903. doi: 10.3390/molecules27154903 (PMC9370425; doi:10.3390/molecules27154903)
Supplement: Supplementary file 1 [file molecules-27-04903-s001.zip › molecules-1834658-supplementary.pdf]

Supporting Information

Two new lactones from leaves of *Ardisia crenata* Sims with antibacterial and anti-inflammatory activities

Huihui Tao, Yongqiang Zhou*, Xin Yin, Xin Wei and Ying Zhou*

College of Pharmacy, Guizhou University of Traditional Chinese Medicine, Guiyang 550025, P. R. China

Correspondence

Doctor Yongqiang Zhou; Professor Ying Zhou
College of Pharmacy, Guizhou University of Traditional Chinese Medicine
Dongqing Road, Huaxi District, Guiyang 550025, P.R. China
E-mail: zhouxiaoqiang1988@126.com; Yingzhou71@sina.com

1 Computational methods **1**
1.1 Conformational analysis **1**
Figure S1 Chemical structure of compounds **1**. 1
Table S1 Energies of configurations **1** 1
1.2 ECD calculation **2**
2 Energies and Coordinates **4**
2.1 Energies at B3LYP theory level **4**
Table S2 Energies of configurations **1** at B3LYP/6-311G(d,p) in methanol. 4
2.2 Coordinates at B3LYP theory level **5**
Table S3 Standard orientations of configurations **1** for ECD calculation. 5
3 Figures **8**
Figure S2. ^{1}H NMR spectrum of 1 in CD_3OD (400 MHz) 8
Figure S3. ^{13}C NMR spectrum of 1 in CD_3OD (100 MHz) 9
Figure S4. dept135° spectrum of 1 in CD_3OD (100 MHz) 10
Figure S5. HSQC spectrum of 1 in CD_3OD (100 MHz) 11
Figure S6. HMBC spectrum of 1 in CD_3OD (100 MHz) 12
Figure S7. ^{1}H-^{1}H COSY spectrum of 1 in CD_3OD (400 MHz). 13
Figure S8. IR spectrum of 1. 14
Figure S9. UV spectrum of 1. 15
Figure S10. ORD spectrum of 1 16
Figure S11. HRESIMS spectrum of 1. 17
Figure S12. ^{1}H NMR spectrum of 2 in CD_3OD (400 MHz) 18
Figure S13. ^{13}C NMR spectrum of 2 in CD_3OD (100 MHz) 19
Figure S14. dept135° spectrum of 2 in CD_3OD (100 MHz) 20
Figure S15. HSQC spectrum of 2 in CD_3OD (100 MHz) 21
Figure S16. HMBC spectrum of 2 in CD_3OD (100 MHz) 22
Figure S17. ^{1}H-^{1}H COSY spectrum of 2 in CD_3OD (400 MHz). 23
Figure S18. IR spectrum of 2. 24
Figure S19. UV spectrum of 2. 25
Figure S20. ORD spectrum of 2 26
Figure S21. HRESIMS spectrum of 2. 27
Table S4 ^{13}C-NMR and ^{1}H-NMR Data of 3-4 in CD_3OD 28
Table S5 ^{13}C-NMR and ^{1}H-NMR Data of 5-6 in CD_3OD 29
Table S6 Effects of ardisicreolide A and B on NO release of raw cells 30
Table S7 The effects of ardisicreolide A and B on production release of TNF-α, IL-1β, IL-4 and IL-10 31
References 32

1 Computational methods

1.1 Conformational analysis

Conformational analysis for compound 1 (**Figure S1**) were performed using systematic algorithm by Confab [1-3] at MMFF94 force field with RMSD threshold of 0.2 Å and energy window of 7 kcal/mol. The detailed experimental process is consistent with our previous work[2-3]. The energies of all dominative conformers were provided in **Table S1**.

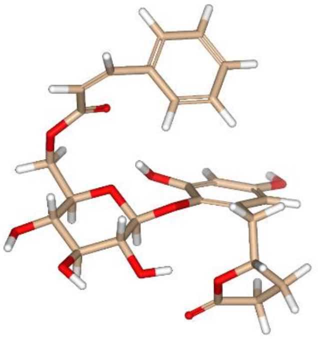

Figure S1 Chemical structure of compounds **1**.

Table S1 Energies of configurations **1**.

Configuration	Conformer	Structure	Energy (kcal/mol)	Population (%)
1	1a	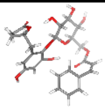	110.4	38
1	1b	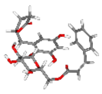	110.58	28
1	1c	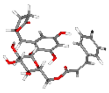	111.05	12.7
1	1d	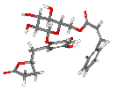	111.54	5.5

1	1e	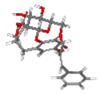	111.94	2.8
1	1f	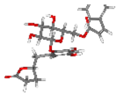	112.08	2.2
1	1g	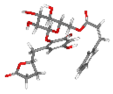	112.18	1.9
1	1h	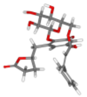	112.3	1.5
1	1i	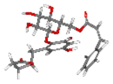	112.51	1.1
1	1j	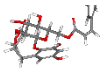	112.55	1

1.2 ECD calculation

The theoretical calculations were carried out using Gaussian 09 [4]. The detailed experimental process is consistent with our previous work[2-3]. At first, all conformers were optimized at PM6. Room-temperature equilibrium populations were calculated according to Boltzmann distribution law **(eq. 1)**, based on which dominative conformers of population over 5% were kept. The chosen conformers were further optimized at B3LYP/6-31G(d,p) in gas phase **(Tables S2 and S3)**. Vibrational frequency analysis confirmed the stable structures. ECD calculations were conducted at B3LYP/6-311G(d,p) level in methanol with IEFPCM model using Time-dependent Density functional theory (TD-DFT). Rotatory strengths for 30 excited states were calculated. The ECD spectrum was simulated using the ECD/UV analysis tool by overlapping Gaussian functions for each transition according to **(eq. 2)**.

$$\frac{N_i}{N} = \frac{g_i e^{-\frac{E_i}{k_B T}}}{\sum g_i e^{-\frac{E_i}{k_B T}}} \quad \textbf{(1)}$$

where N_i is the number of conformer i with energy E_i and degeneracy g_i at temperature *T*, and k_B is Boltzmann constant.

$$\Delta\varepsilon(E) = \frac{1}{2.297\times10^{-39}} \times \frac{1}{\sqrt{2\pi\sigma}} \sum_{i}^{A} \Delta E_i R_i e^{-\left(\frac{E-E_i}{2\sigma}\right)^2} \qquad \textbf{(2)}$$

where σ represents the width of the band at 1/*e* height, while ΔE_i and R_i are the excitation energies and rotatory strengths for transition i, respectively.

The σ and UV-shift values were set 0.27 eV and -13 nm, respectively. The spectrum of the enantiomers were produced directly by mirror inversion about the horizontal axis.

2 Energies and Coordinates

2.1 Energies at B3LYP theory level

Structures for ECD calculation were shown in **Table S**2.

Table S2 Energies of configurations **1** at B3LYP/6-311G(d,p) in methanol.

Configuration	Conformer	Structure	E (Hartree)	E (kcal/mol)	Population (%)
1	1e	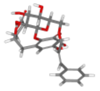	-1835.589597	-1151849.85	78.16
1	1j	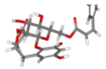	-1835.588394	-1151849.1	21.84

2.2 Coordinates at B3LYP theory level

Table S3 Standard orientations of configurations **1** for ECD calculation.

Conformer **1**-1e

Center	Atomic	Atomic	Coordinates (Angstroms)		
Number	Number	Type	X	Y	Z
1	6	0	-2.781397	-0.712892	-1.818198
2	8	0	-2.232896	0.053708	0.310402
3	6	0	-2.288897	-1.077892	-0.478098
4	8	0	-1.941497	-2.159092	-0.055798
5	8	0	0.925804	-0.209593	0.147102
6	6	0	2.026203	-1.020594	0.431302
7	6	0	2.859203	-1.435994	-0.628598
8	6	0	3.824403	-2.415694	-0.381798
9	6	0	3.964403	-2.944894	0.913802
10	6	0	3.169503	-2.528194	1.988002
11	6	0	2.195003	-1.554694	1.733602
12	8	0	1.445503	-1.175793	2.797102
13	8	0	4.937603	-3.890494	1.049802
14	6	0	2.695003	-0.863594	-2.005098
15	6	0	3.700104	0.233406	-2.358198
16	8	0	3.422204	1.368106	-1.449598
17	6	0	4.458104	1.544406	-0.535798
18	6	0	5.610104	0.646605	-0.877198
19	6	0	5.189304	-0.095995	-2.150598
20	8	0	4.277504	2.345506	0.349202
21	6	0	-4.069397	-0.651992	-2.171698
22	6	0	-5.219697	-0.893391	-1.283898
23	6	0	-5.316396	-0.265991	-0.031898
24	6	0	-6.429196	-0.497191	0.780902
25	6	0	-7.445797	-1.356591	0.352802
26	6	0	-7.352497	-1.982491	-0.895298
27	6	0	-6.247797	-1.748691	-1.716998
28	6	0	-1.043396	1.252907	2.029802
29	6	0	-1.645796	2.458307	1.282802
30	6	0	-0.704495	3.043507	0.213102
31	6	0	0.171804	1.960407	-0.454298
32	6	0	0.984604	1.162807	0.585002
33	8	0	0.397604	1.180807	1.892702
34	8	0	1.055304	2.728507	-1.274298
35	8	0	-1.589795	3.591607	-0.774598
36	8	0	-1.837795	3.432508	2.322202
37	6	0	-1.699196	-0.080793	1.663102
38	1	0	-1.973796	-0.517992	-2.536098
39	1	0	4.471303	-2.780494	-1.179298
40	1	0	3.287603	-2.936494	2.988602
41	1	0	0.817204	-0.391893	2.564702
42	1	0	4.996802	-4.255595	1.980202
43	1	0	2.763403	-1.673194	-2.769898
44	1	0	1.661104	-0.455594	-2.118598
45	1	0	3.488804	0.657506	-3.365198
46	1	0	6.544604	1.231005	-1.018598
47	1	0	5.829504	-0.050995	-0.039798
48	1	0	5.788404	0.232605	-3.022598
49	1	0	5.368503	-1.185695	-2.069498

50	1	0	-4.337796	-0.417292	-3.213998
51	1	0	-4.523896	0.406708	0.304302
52	1	0	-6.503296	-0.006191	1.751902
53	1	0	-8.311997	-1.537891	0.990002
54	1	0	-8.145797	-2.652291	-1.229298
55	1	0	-6.181797	-2.234691	-2.691298
56	1	0	-1.114496	1.427807	3.142402
57	1	0	-2.639096	2.212908	0.827202
58	1	0	-0.072595	3.864407	0.627002
59	1	0	-0.435296	1.276607	-1.096198
60	1	0	2.024804	1.517806	0.755202
61	1	0	1.721204	2.138306	-1.728998
62	1	0	-1.058295	4.024107	-1.490898
63	1	0	-2.423795	4.160508	2.000502
64	1	0	-0.972597	-0.919293	1.694702
65	1	0	-2.567796	-0.309592	2.310402

Conformer **1**-1j

Center Number	Atomic Number	Atomic Type	Coordinates (Angstroms)		
			X	Y	Z
1	6	0	-4.134402	-1.791591	-1.865899
2	8	0	-2.331196	-0.317299	-1.849599
3	6	0	-3.708996	-0.384993	-1.971799
4	8	0	-4.366892	0.613710	-2.158999
5	8	0	1.017607	0.367987	-1.002399
6	6	0	1.586301	-0.881916	-0.746599
7	6	0	2.956101	-1.069021	-1.024899
8	6	0	3.497395	-2.350124	-0.896199
9	6	0	2.677291	-3.410220	-0.468899
10	6	0	1.317791	-3.245914	-0.177599
11	6	0	0.773697	-1.963812	-0.328399
12	8	0	-0.544503	-1.839507	-0.039399
13	8	0	3.302986	-4.617823	-0.359099
14	6	0	3.796605	0.083275	-1.488899
15	6	0	4.796208	0.608071	-0.459699
16	8	0	4.021409	1.011074	0.735401
17	6	0	4.374406	0.251973	1.847401
18	6	0	5.482302	-0.698532	1.502101
19	6	0	5.850803	-0.393234	0.047001
20	8	0	3.775007	0.463375	2.873201
21	6	0	-4.539605	-2.370490	-0.731099
22	6	0	-4.582102	-1.716689	0.589001
23	6	0	-5.760702	-1.786784	1.350801
24	6	0	-5.815900	-1.175784	2.605601
25	6	0	-4.696897	-0.505989	3.112801
26	6	0	-3.519497	-0.444694	2.360701
27	6	0	-3.460799	-1.045194	1.100701
28	6	0	-1.684188	1.624298	-0.558899
29	6	0	-1.419582	3.142997	-0.616999
30	6	0	-0.136880	3.566192	0.125601
31	6	0	1.030016	2.590987	-0.134999
32	6	0	0.635310	1.130288	0.164601
33	8	0	-0.770591	0.927294	0.318401
34	8	0	2.019118	2.994183	0.816201
35	8	0	0.188226	4.842390	-0.442999
36	8	0	-2.559779	3.692302	0.063801
37	6	0	-1.691191	0.983498	-1.950099

38	1	0	-4.111505	-2.329691	-2.822499
39	1	0	4.547594	-2.540728	-1.118799
40	1	0	0.693288	-4.071312	0.154401
41	1	0	-0.900399	-0.884905	-0.216799
42	1	0	2.689083	-5.341820	-0.041199
43	1	0	4.350104	-0.200627	-2.415799
44	1	0	3.132209	0.923778	-1.806599
45	1	0	5.248512	1.566569	-0.800899
46	1	0	6.345303	-0.575636	2.190501
47	1	0	5.152098	-1.752031	1.637201
48	1	0	6.868605	0.036962	-0.028799
49	1	0	5.877100	-1.315234	-0.568199
50	1	0	-4.887109	-3.415088	-0.739599
51	1	0	-6.634305	-2.311481	0.961601
52	1	0	-6.734200	-1.223980	3.192701
53	1	0	-4.742595	-0.034589	4.095101
54	1	0	-2.644495	0.071402	2.757901
55	1	0	-2.534799	-1.000198	0.518301
56	1	0	-2.655489	1.434602	-0.020399
57	1	0	-1.402480	3.543197	-1.660699
58	1	0	-0.313679	3.689393	1.220301
59	1	0	1.433717	2.698285	-1.169999
60	1	0	1.057708	0.720787	1.107301
61	1	0	2.883616	2.516179	0.643201
62	1	0	0.978827	5.219587	0.021001
63	1	0	-2.562675	4.678102	-0.021799
64	1	0	-2.213588	1.599801	-2.705999
65	1	0	-0.672492	0.717594	-2.309599

3 Figures

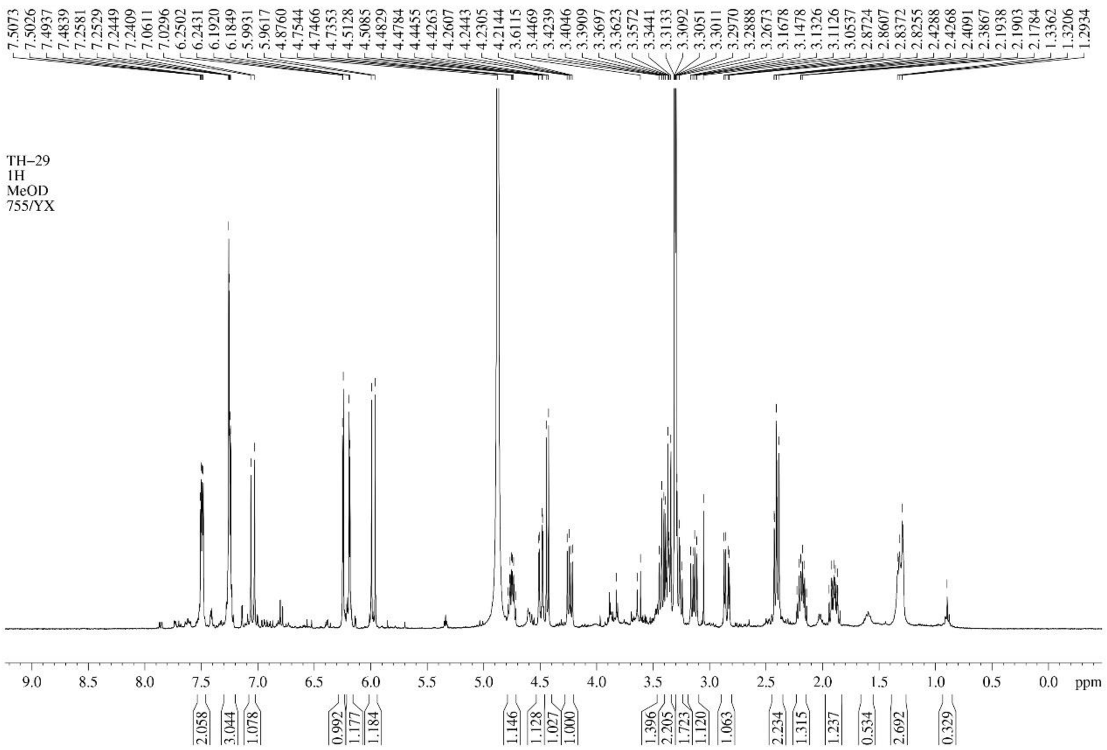

Figure S2. ^{1}H NMR spectrum of 1 in CD_3OD (400 MHz)

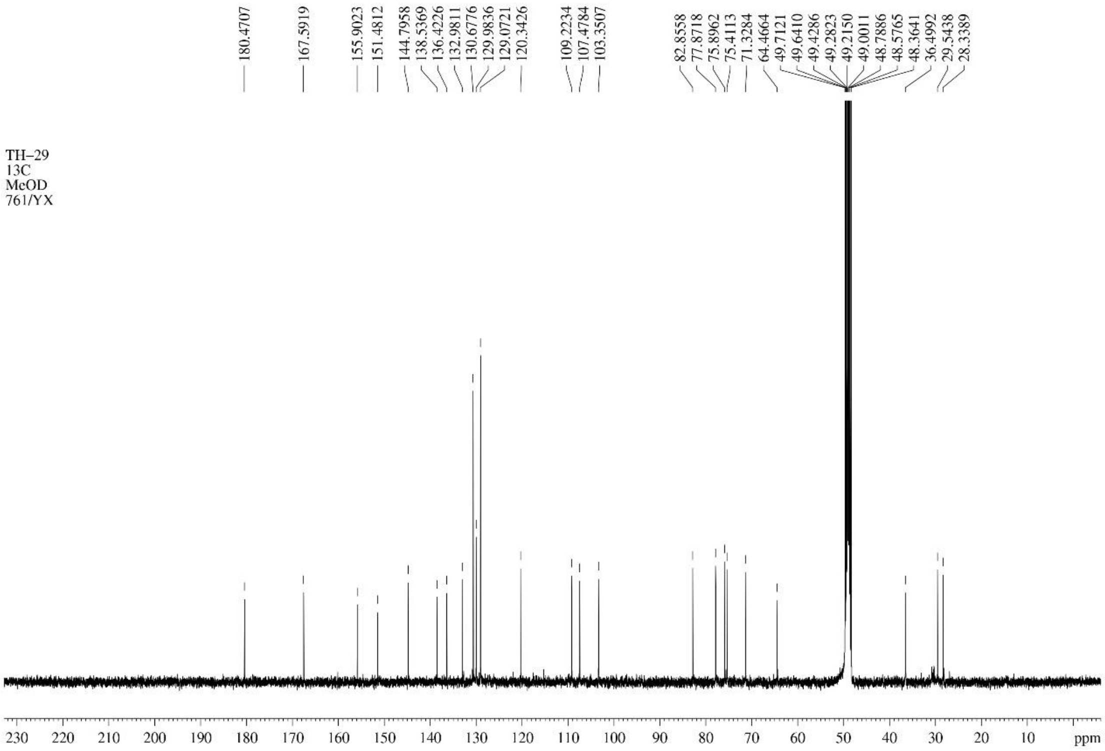

Figure S3. ^{13}C NMR spectrum of 1 in CD_3OD (100 MHz)

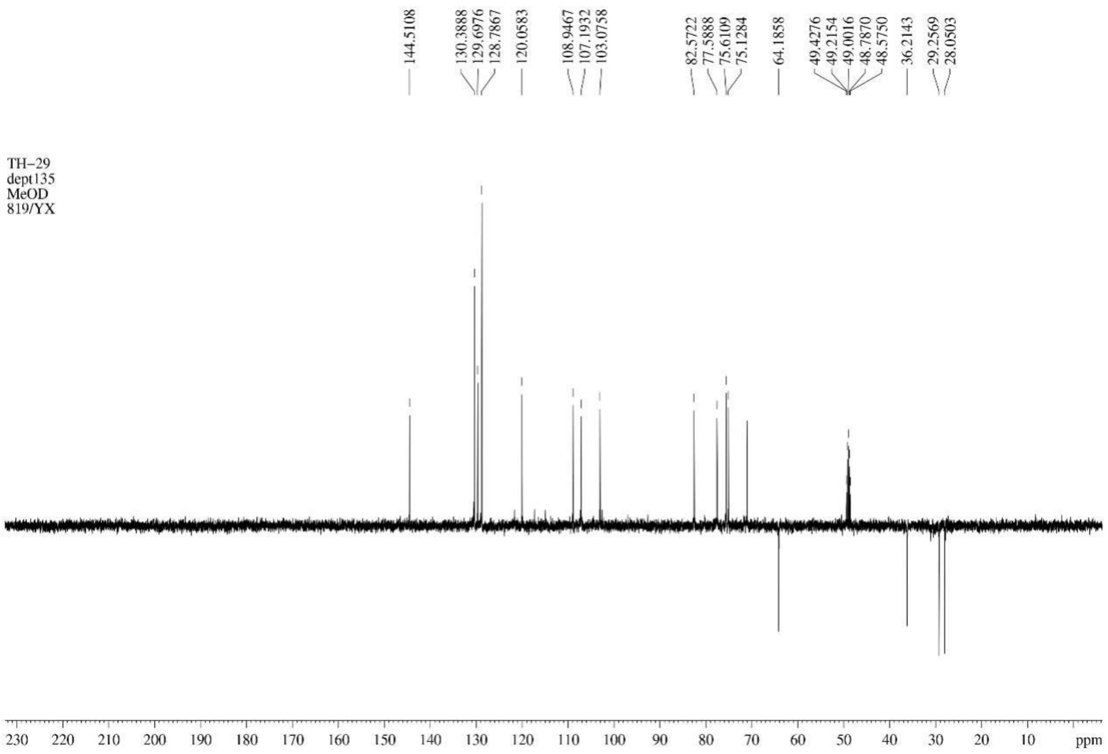

Figure S4. dept135° spectrum of 1 in CD_3OD (100 MHz)

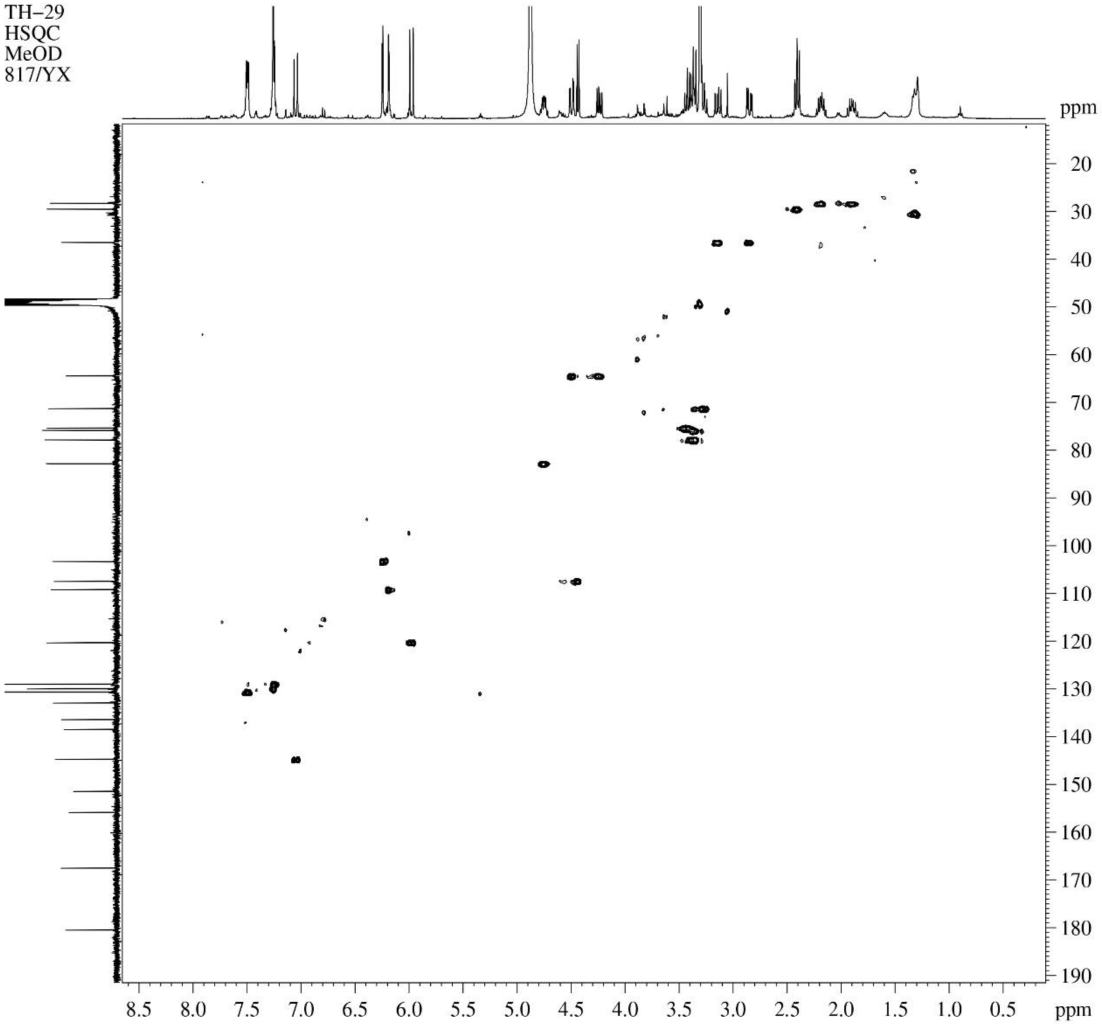

Figure S5. HSQC spectrum of 1 in CD_3OD (100 MHz)

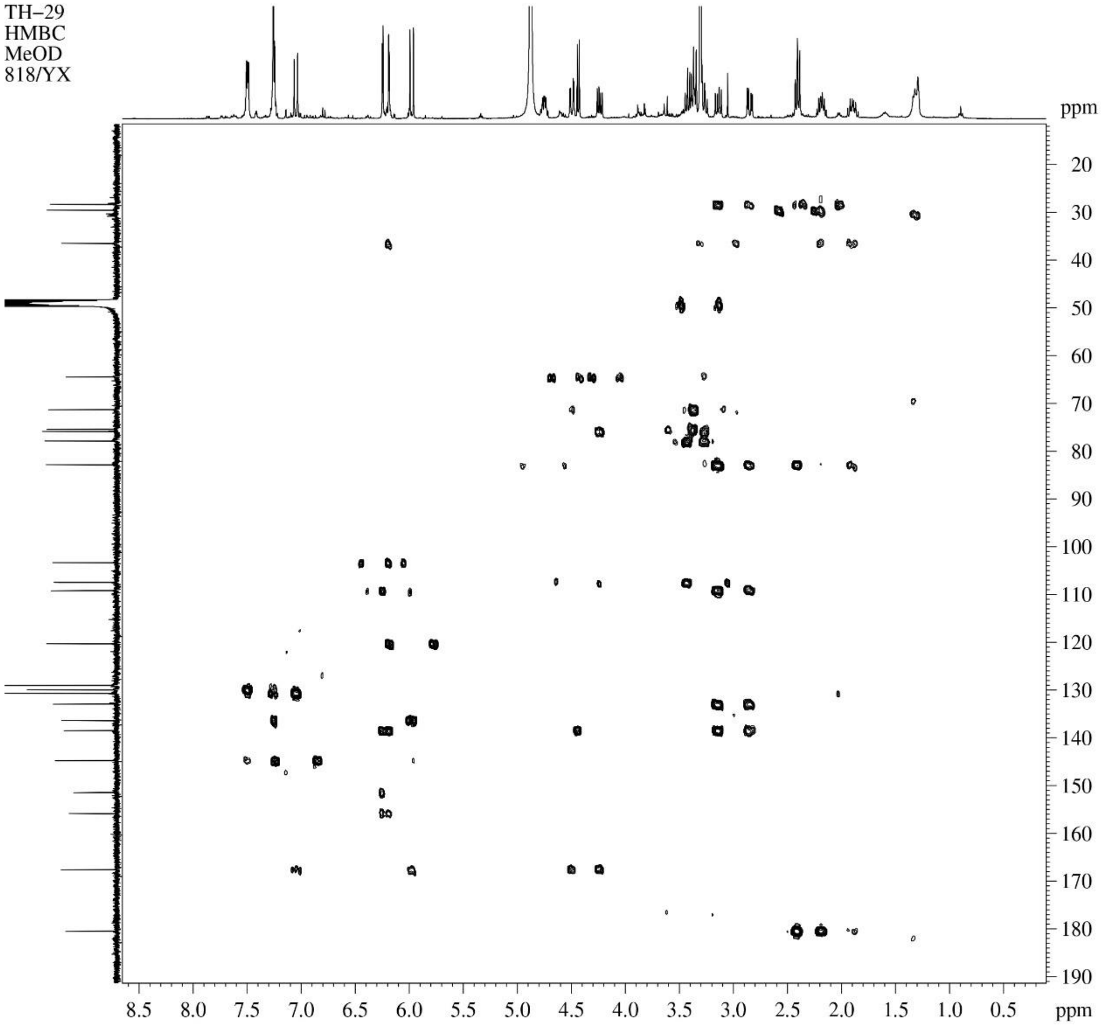

Figure S6. HMBC spectrum of 1 in CD_3OD (100 MHz)

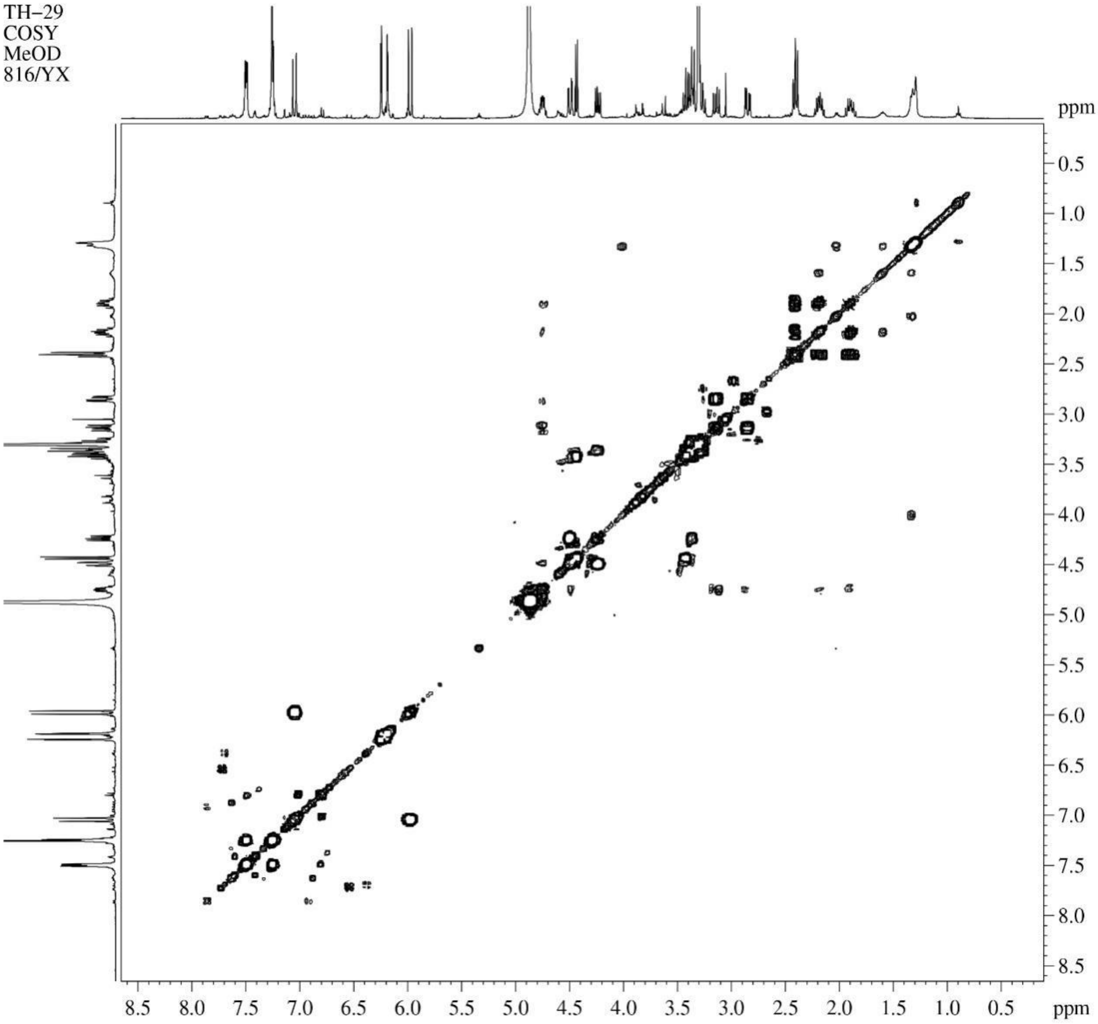

Figure S7. ^{1}H-^{1}H COSY spectrum of 1 in CD_3OD (400 MHz).

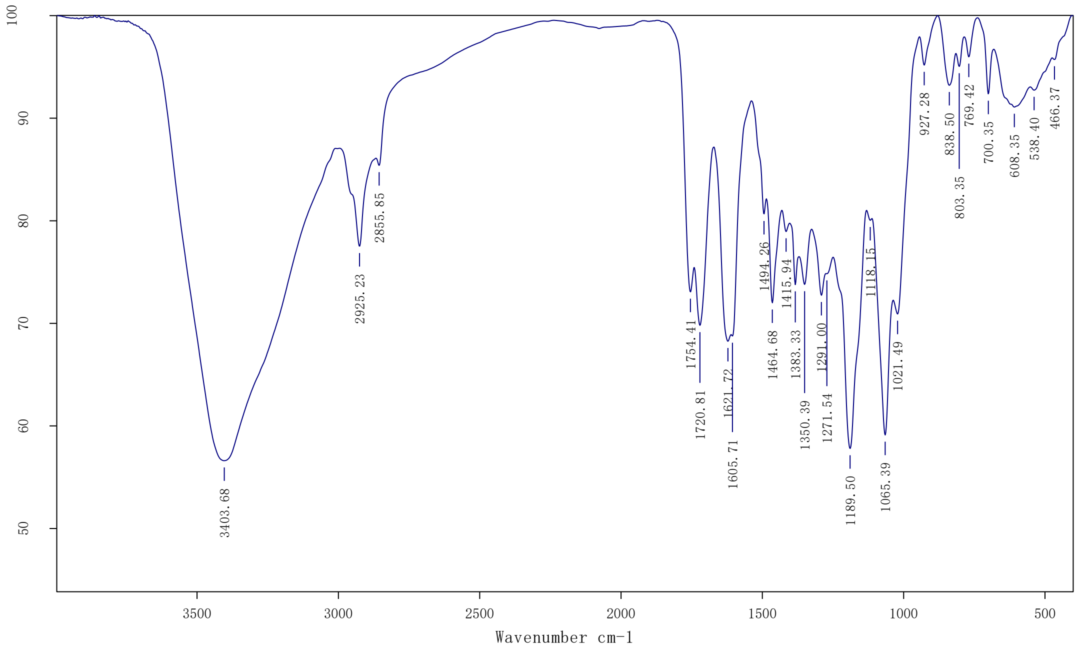

Figure S8. IR spectrum of 1.

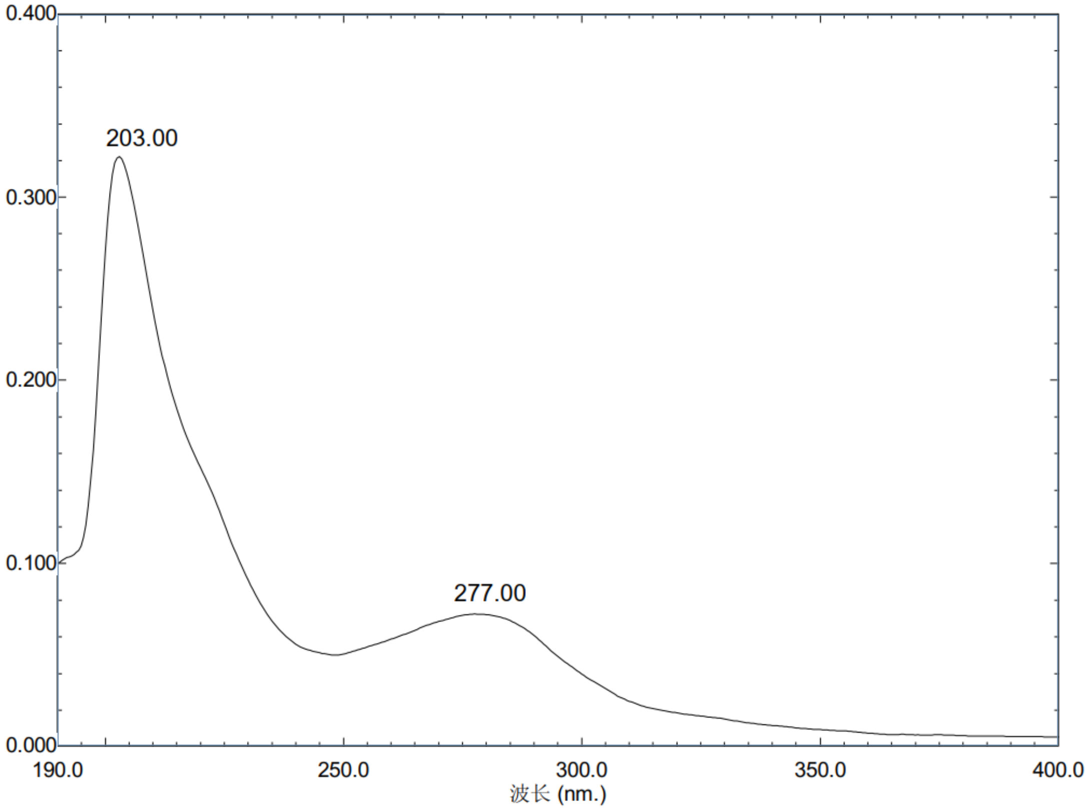

Figure S9. UV spectrum of 1.

Rudolph Research Analytical

This sample was measured on an Autopol VI, Serial #91058
Manufactured by Rudolph Research Analytical, Hackettstown, NJ, USA.

Measurement Date : Wednesday, 15-DEC-2021

Set Temperature : OFF

Time Delay : Disabled

Delay between Measurement : Disabled

n	Average	Std.Dev.	% RSD	Maximum	Minimum
5	14.23	0.45	3.16	14.62	13.65

S.No	Sample ID	Time	Result	Scale	OR °Arc	WLG.nm	Lg.mm	Conc.g/100ml	Temp.
1	TH-29	03:21:27 PM	13.65	SR	0.0071	589	100.00	0.052	19.6
2	TH-29	03:21:35 PM	14.62	SR	0.0076	589	100.00	0.052	19.6
3	TH-29	03:21:43 PM	14.42	SR	0.0075	589	100.00	0.052	19.6
4	TH-29	03:21:51 PM	14.62	SR	0.0076	589	100.00	0.052	19.6
5	TH-29	03:22:00 PM	13.85	SR	0.0072	589	100.00	0.052	19.6

Figure S10. ORD spectrum of 1.

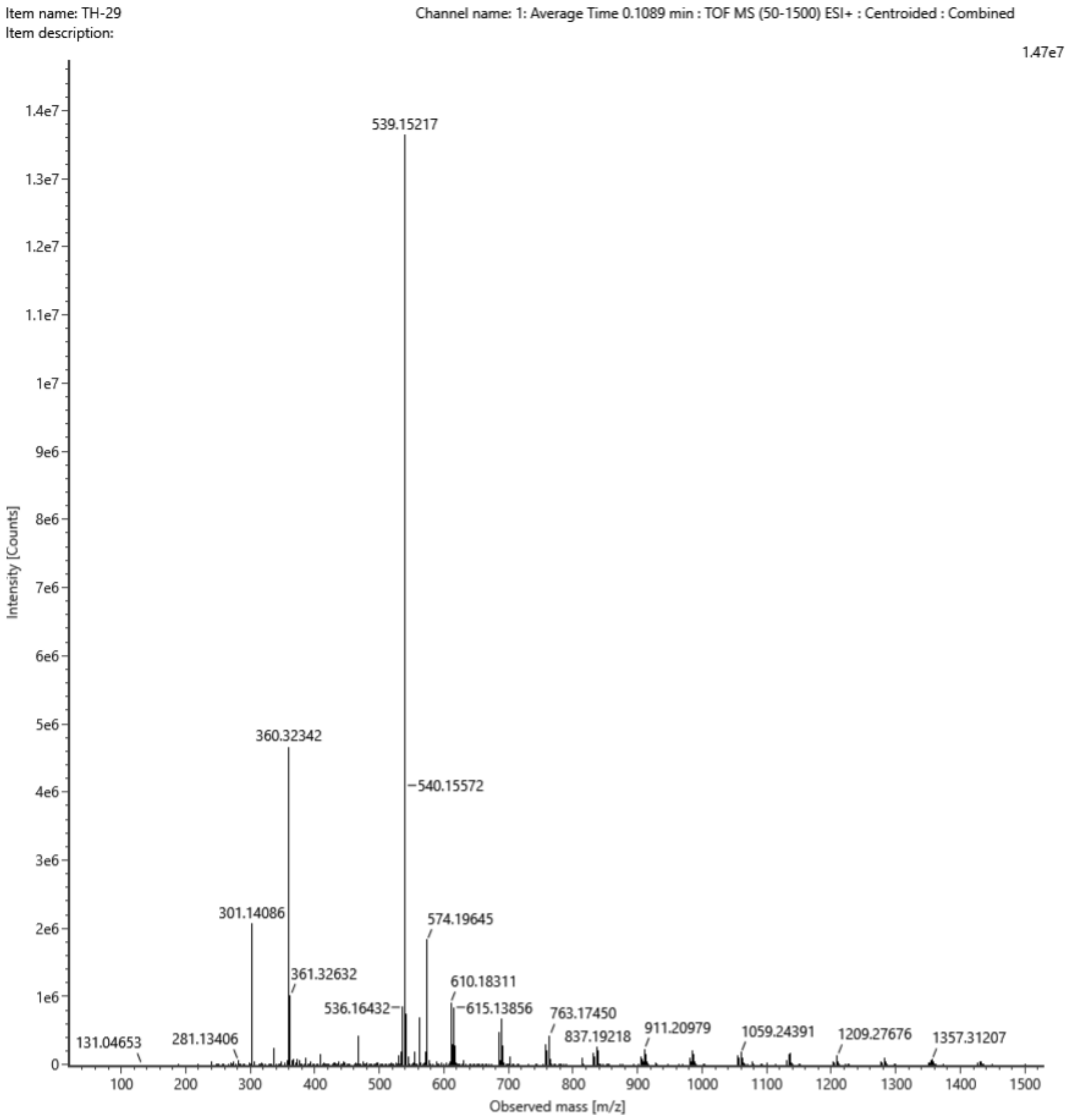

Add:Na^+

Composition	i-FIT Confidence (%)	Predicted m/z	m/z error (PPM)
$C_{26}H_{28}O_{11}$	100.000000	539.152383	-0.395467

Figure S11. HRESIMS spectrum of 1.

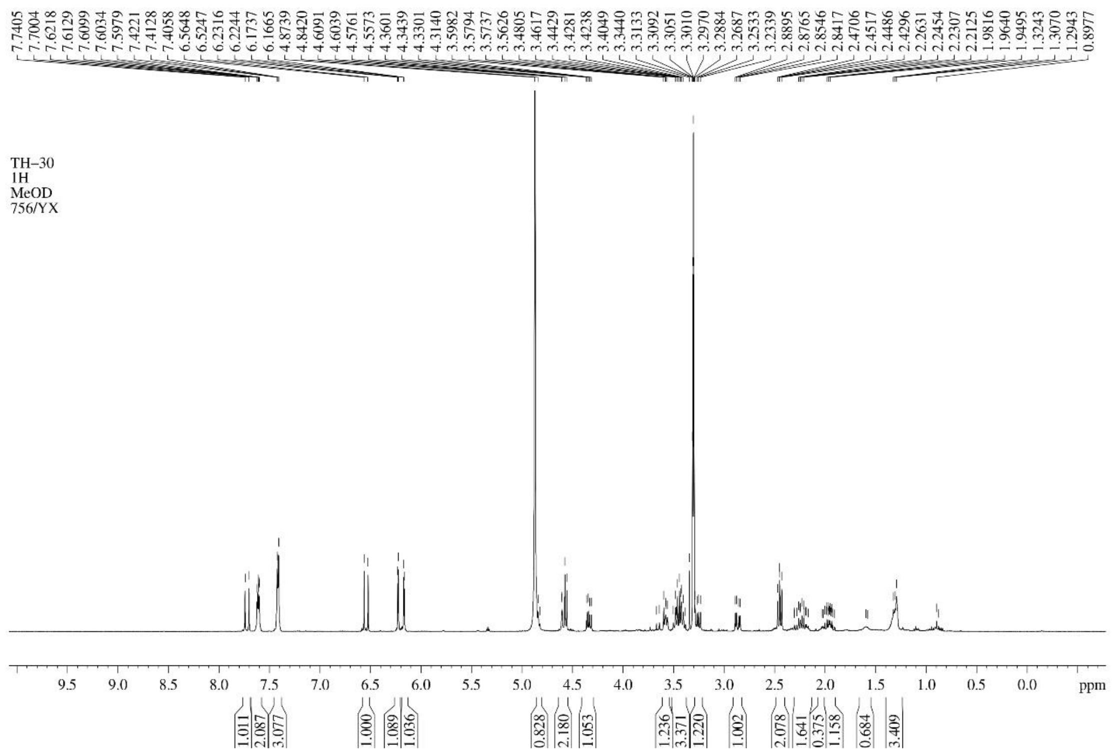

Figure S12. ^{1}H NMR spectrum of 2 in CD_3OD (400 MHz)

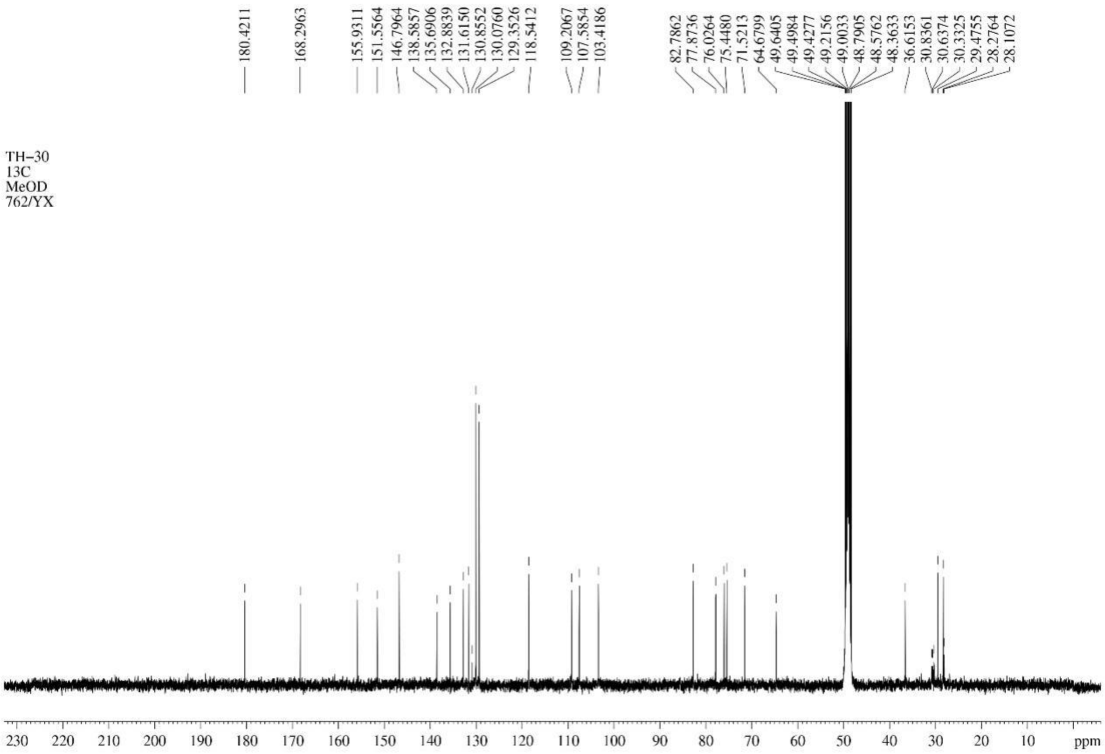

Figure S13. ^{13}C NMR spectrum of 2 in CD_3OD (100 MHz)

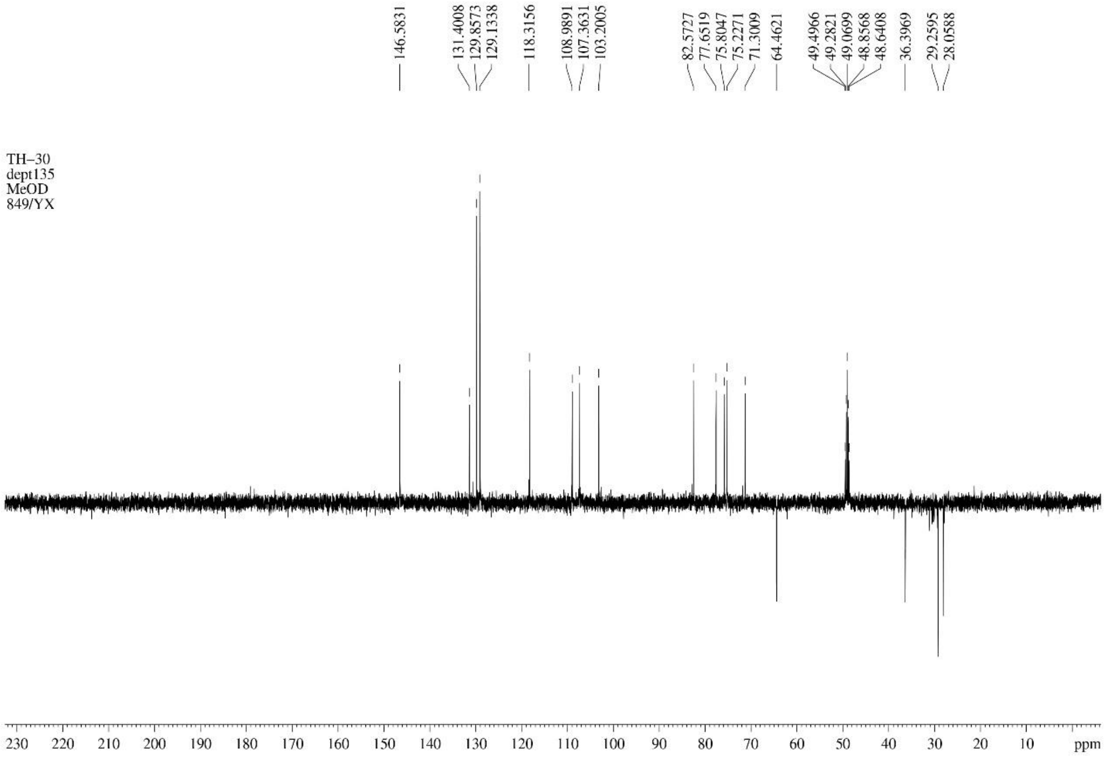

Figure S14. dept135° spectrum of 2 in CD_3OD (100 MHz)

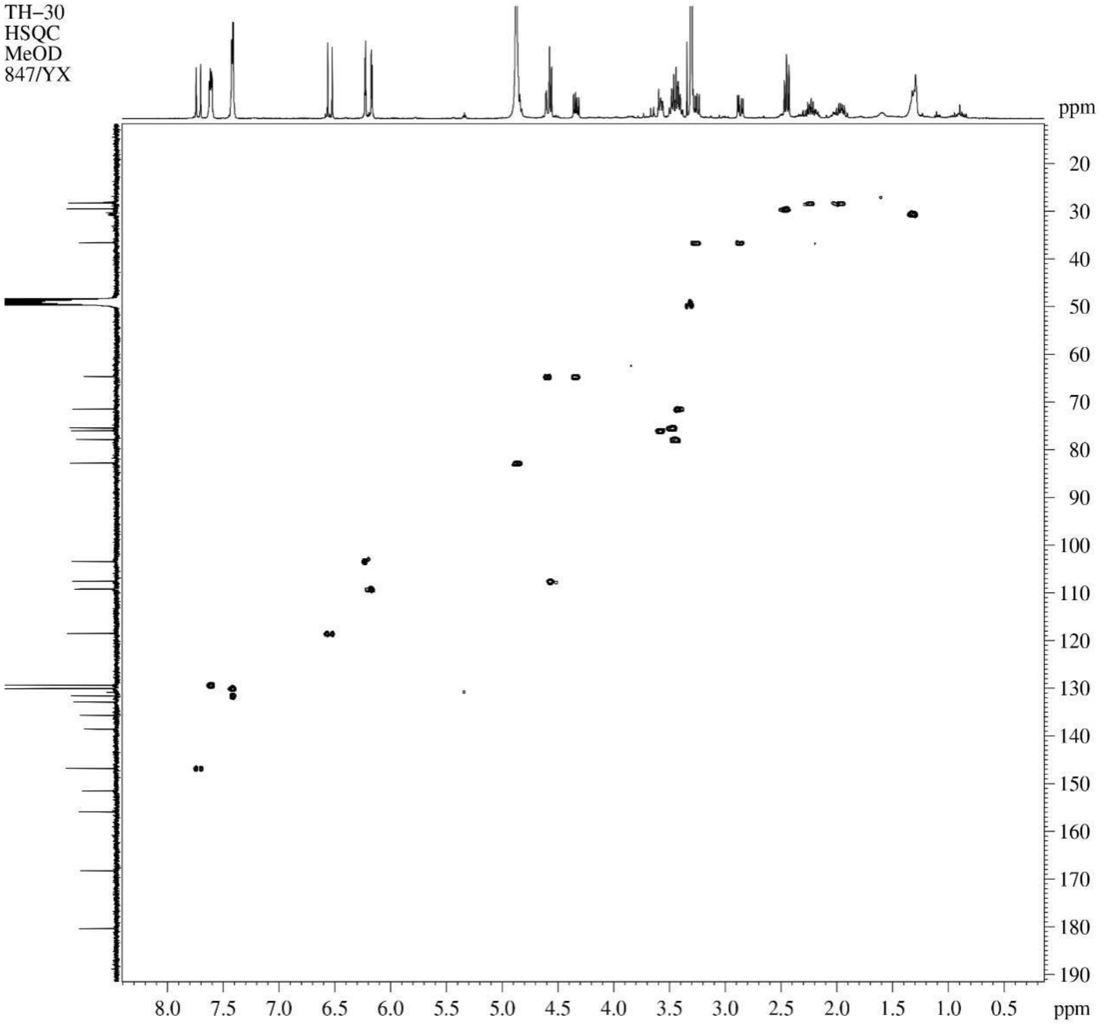

Figure S15. HSQC spectrum of 2 in CD_3OD (100 MHz)

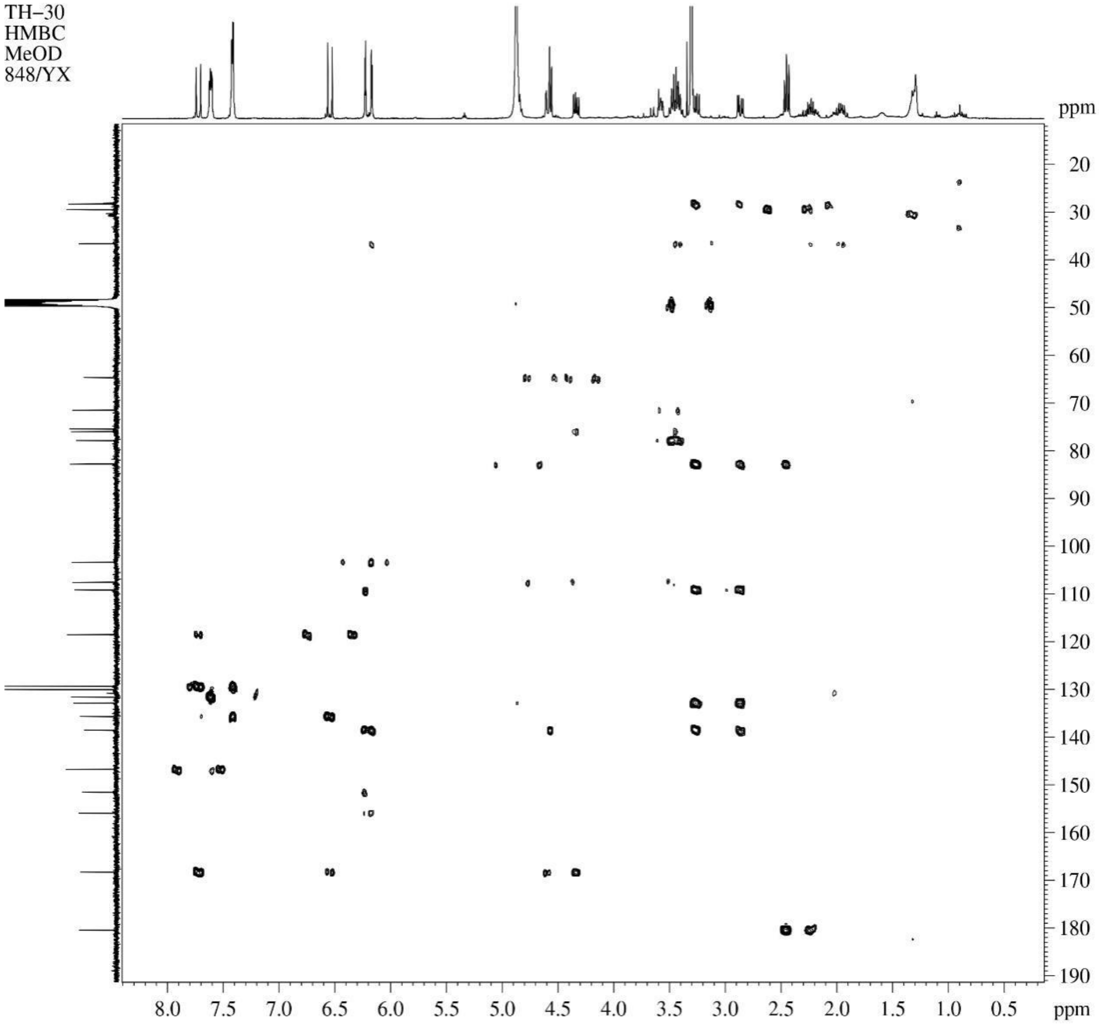

Figure S16. HMBC spectrum of 2 in CD_3OD (100 MHz)

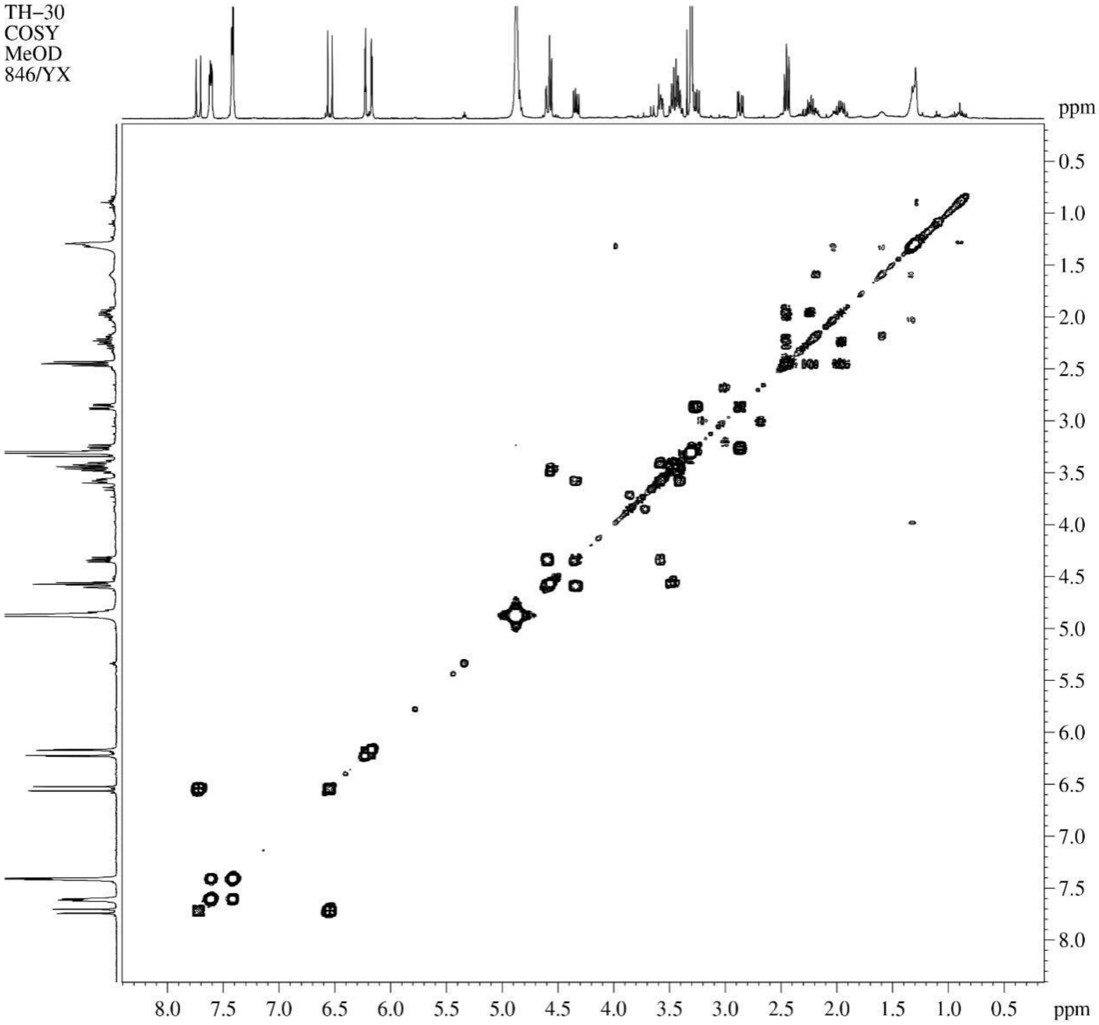

Figure S17. ^{1}H-^{1}H COSY spectrum of 2 in CD_3OD (400 MHz).

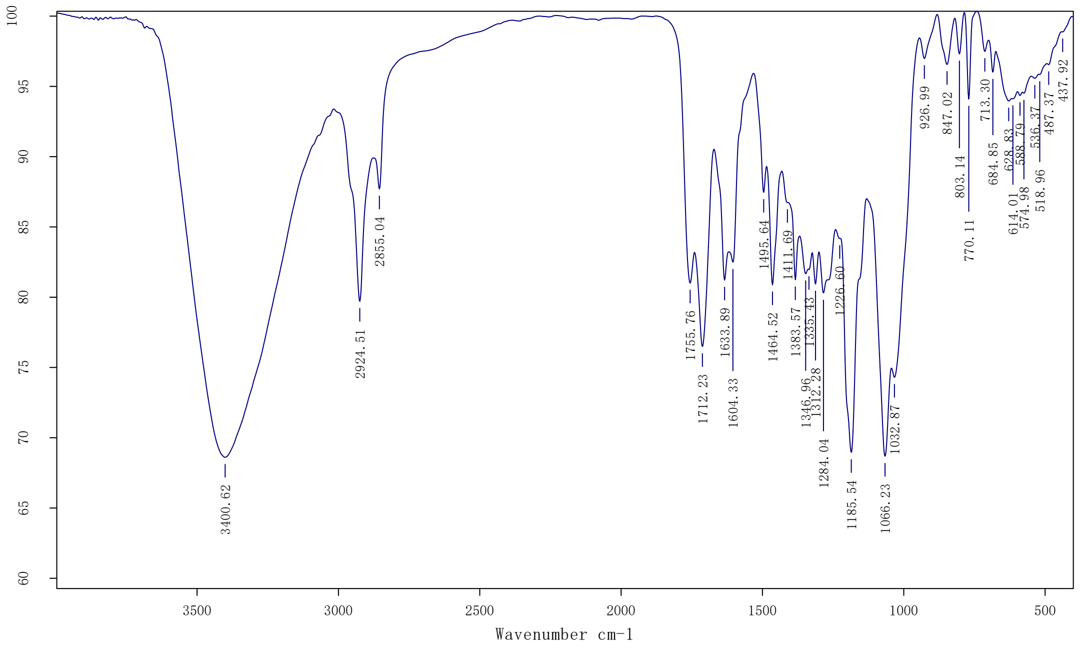

Figure S18. IR spectrum of 2.

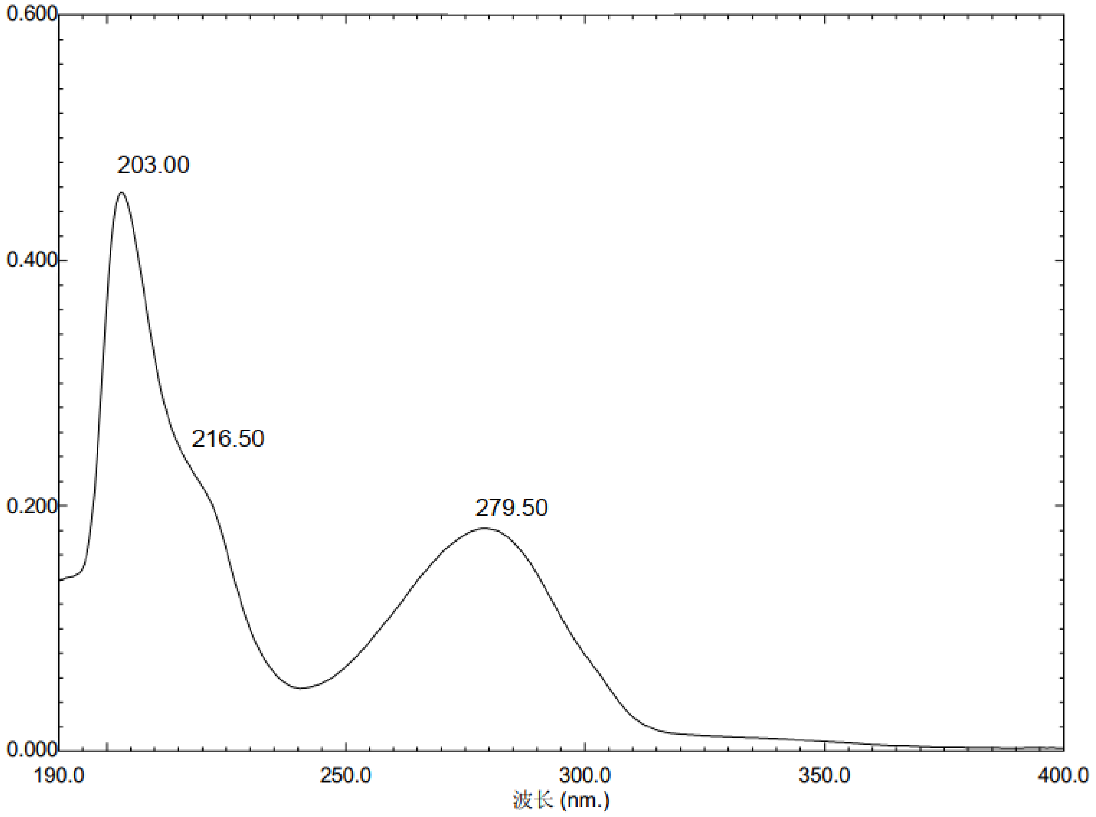

Figure S19. UV spectrum of 2.

Rudolph Research Analytical

This sample was measured on an Autopol VI, Serial #91058
Manufactured by Rudolph Research Analytical, Hackettstown, NJ, USA.

Measurement Date : Wednesday, 15-DEC-2021

Set Temperature : OFF

Time Delay : Disabled

Delay between Measurement : Disabled

n	Average	Std.Dev.	% RSD	Maximum	Minimum
5	-22.65	1.03	-4.54	-21.57	-23.98

S.No	Sample ID	Time	Result	Scale	OR °Arc	WLG.nm	Lg.mm	Conc.g/100ml	Temp.
1	TH-30	03:39:26 PM	-23.98	SR	-0.0199	589	100.00	0.083	19.7
2	TH-30	03:39:34 PM	-23.25	SR	-0.0193	589	100.00	0.083	19.7
3	TH-30	03:39:43 PM	-22.77	SR	-0.0189	589	100.00	0.083	19.7
4	TH-30	03:39:51 PM	-21.69	SR	-0.0180	589	100.00	0.083	19.7
5	TH-30	03:39:59 PM	-21.57	SR	-0.0179	589	100.00	0.083	19.7

Figure S20. ORD spectrum of 2.

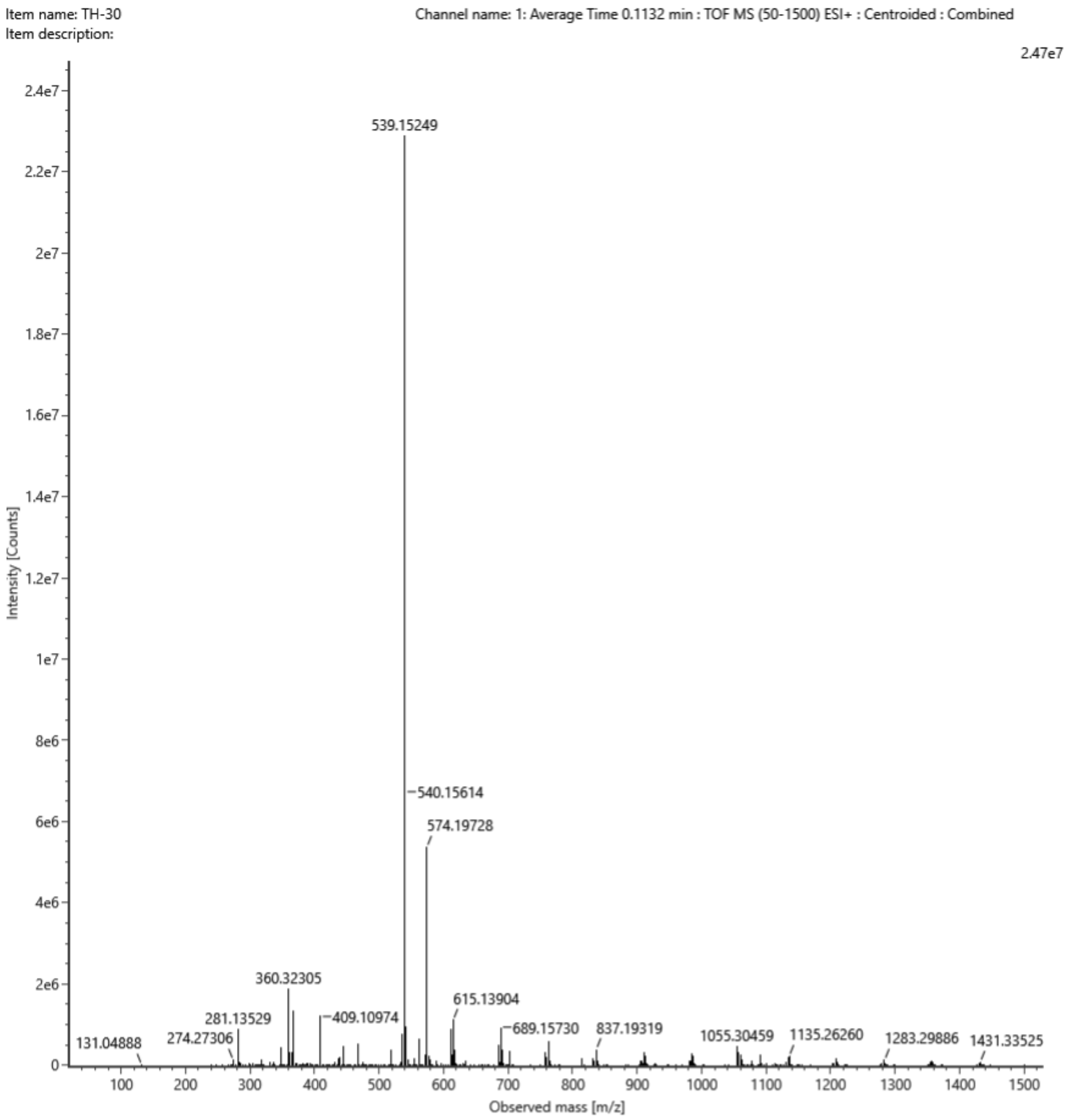

Add:Na+

Composition	i-FIT Confidence (%)	Predicted m/z	m/z error (PPM)
$C_{26}H_{28}O_{11}$	100.000000	539.152383	0.199168

Figure S21. HRESIMS spectrum of 2.

Table S4 ^{13}C-NMR (100 MHz) and ^{1}H-NMR (400 MHz) Data of 3-4 in CD_3OD

	Myricetrin (3)		Quercitrin (4)	
No	C	H	C	H
2	159.4	-	158.4	-
3	136.3	-	136.2	-
4	179.7	-	179.6	-
5	163.2	-	159.2	-
6	99.8	6.20 (1H, d, *J* = 2.1 Hz)	99.8	6.18 (1H, s),
7	165.9	-	165.8	-
8	94.7	6.36 (1H, d, *J* = 2.1 Hz)	94.7	6.34 (1H, s)
9	158.5	-	163.1	-
10	105.9	-	105.8	-
1'	121.9	-	122.9	-
2'	109.6	6.95 (2H, s)	116.3	7.22 – 7.37 (2H, m)
3'	146.9	-	146.3	-
4'	109.6	-	149.7	-
5'	146.9	-	116.9	6.90 (1H, d, *J* = 8.3 Hz)
6'	109.6	6.95 (s)	122.9	7.22 – 7.37 (2H, m).
1''	103.6	5.31 (1H, d, *J* = 1.7 Hz)	103.5	5.34 (1H, d, *J* = 1.5 Hz)
2''	71.9	4.22 (1H, dd, *J* = 1.7, 3.4 Hz)	72.0	4.23-3.37 (sugar H)
3''	72.1	3.79 (1H, dd, *J* = 3.4, 9.5 Hz)	72.1	-
4''	73.3	3.51 (1H, dd, *J* = 2.6, 7.0 Hz)	73.2	-
5''	72.0	3.51 – 3.69 (1H, m)	71.9	-
6''	17.7	0.96 (3H, d, *J* = 6.2 Hz)	17.6	0.94 (3H, d, *J* = 6.0 Hz)

Table S5 ^{13}C-NMR (100 MHz) and ^{1}H-NMR (400 MHz) Data of 5-6 in CD_3OD

	Quercetin（5）		Tamarixetin-3-O-rhamnoside (6)	
No	C	H	C	H
2	148.0	-	158.9	-
3	137.2	-	136.4	-
4	177.3	-	179.6	-
5	162.5	-	158.6	-
6	99.2	6.17 (1H, s)	94.7	6.38 (1H, d, *J* = 2.1 Hz)
7	165.6	-	166.0	-
8	94.4	6.38 (1H, s)	99.9	6.20 (1H, d, *J* = 2.1 Hz)
9	158.2	-	163.2	-
10	104.5	-	105.9	-
1’	124.2	-	124.2	-
2’	116.0	7.73 (1H, d, *J* = 2.1 Hz)	116.6	7.34 (1H, d, *J* = 2.1 Hz)
3’	146.2	-	147.7	-
4’	148.8	-	151.6	-
5’	116.2	6.88 (1H, d, *J* = 8.4 Hz)	112.3	7.07 (1H, d, *J* = 8.5 Hz)
6’	121.7	7.62 (1H, dd, *J* = 8.5, 1.7 Hz)	122.7	7.41 (1H, dd, *J* = 8.5, 2.2 Hz)
Rha-1	-	-	103.5	5.37 (1H, d, *J* = 1.7 Hz)
2	-	-	71.9	4.21 (1H, dd, *J* = 1.7, 3.4 Hz)
3	-	-	72.0	3.73 (1H, s)
4	-	-	72.1	3.35 (2H, m)
5	-	-	73.2	
6	-	-	17.7	0.92 (3H, d, *J* = 5.6 Hz)
$C_{4'}$-Me	-	-	56.4	3.94 (3H, s)

Table S6. Effects of ardisicreolide A and B on NO release of raw cells

Compound	C(μM/mL)	Mean±SD(μM/mL)
Blank	-	12.80±1.97
LPS	-	55.25±6.59
DXM[b]	-	25.88±2.55
	5	37.17±3.11
Ardisicreolide A	20	40.35±3.95
	80	37.88±3.92
	5	35.04±3.47
Ardisicreolide B	20	36.75±2.69
	80	38.99±2.98

[b] Dexamethasone (DXM) with 40μg/ml was used as the positive control.

Table S7. The effects of ardisicreolide A and B on production release of TNF-α, IL-1β, IL-4 and IL-10

**Compound**	**C(μM/mL)**	**Mean±SD(pg/mL)**			
		**TNF-α**	**IL-1β**	**IL-4**	**IL-10**
Blank	-	43.82±6.22	53.06±4.54	32.5±4.88	89.94±10.00
LPS	-	162.34±8.64	192.02±18.67	118.81±6.34	365.33±28.36
DXM[b]	-	53.95±6.06	71.72±6.81	44.60±5.41	128.02±10.72
	5	83.51±6.86	83.16±6.66	73.89±5.37	139.25±12.18
1	20	81.64±7.32	75.21±7.40	67.75±5.58	131.26±11.93
	80	78.18±7.05	69.48±6.35	65.80±5.55	124.07±11.75
	5	93.51±7.65	97.80±8.15	61.20±5.32	152.83±10.94
2	20	90.00±7.39	95.55±8.80	56.18±5.11	146.79±11.35
	80	83.11±6.74	91.06±8.11	53.82±5.37	134.48±11.22

[b] Dexamethasone (DXM) with 40μg/ml was used as the positive control.

References

1. Noel M OBoyle, Tim V, ermeersch, Christopher J Flynn, Anita R Maguire Maguire, and Geoffey R Hutchison. Confab - systematic generation of diverse low-energy conformers. *Journal of Cheminformatics*, 3:3–8, March 2011.
2. Wei X, Huang XT, Zhang LY, Hu XY, Zhang W, Zhou YQ, Yu HF, Ding CF, Zhang LC, Liu X, Zhou Y. New oxindole alkaloids with selective osteoclast inhibitory activity from Gelsemium elegans, Natural Product Research, 2022, 36(10): 2630-2636.
3. Wei X, Guo R, Wang X, Liang JJ, Yu HF, Ding CF, Feng TT, Zhang LY, Liu X, Hu XY, Zhou Y. New Monoterpenoid Indoles with Osteoclast Activities from Gelsemium elegans. Molecules. 2021, 26(24): 7457.
4. M. J. Frisch, G. W. Trucks, H. B. Schlegel, G. E. Scuseria, M. A. Robb, J. R. Cheeseman, G. Scalmani, V. Barone, B. Mennucci, G. A. Petersson, H. Nakatsuji, M. Caricato, X. Li, H. P. Hratchian, A. F. Izmaylov, J. Bloino, G. Zheng, J. L. Sonnenberg, M. Hada, M. Ehara, K. Toyota, R. Fukuda, J. Hasegawa, M. Ishida, T. Nakajima, Y. Honda, O. Kitao, H. Nakai, T. Vreven, J. A. Montgomery, Jr., J. E. Peralta, F. Ogliaro, M. Bearpark, J. J. Heyd, E. Brothers, K. N. Kudin, V. N. Staroverov, R. Kobayashi, J. Normand, K. Raghavachari, A. Rendell, J. C. Burant, S. S. Iyengar, J. Tomasi, M. Cossi, N. Rega, J. M. Millam, M. Klene, J. E. Knox, J. B. Cross, V. Bakken, C. Adamo, J. Jaramillo, R. Gomperts, R. E. Stratmann, O. Yazyev, A. J. Austin, R. Cammi, C. Pomelli, J. W. Ochterski, R. L. Martin, K. Morokuma, V. G. Zakrzewski, G. A. Voth, P. Salvador, J. J. Dannenberg, S. Dapprich, A. D. Daniels, O. Farkas, J. B. Foresman, J. V. Ortiz, J. Cioslowski, and D. J. Fox. Gaussian 09 Revision D.01. Gaussian Inc. Wallingford CT 2009.
